# Alcohol-Attributable Deaths and Years of Potential Life Lost — 11 States, 2006–2010

**Published:** 2014-03-14

**Authors:** Katherine Gonzales, Jim Roeber, Dafna Kanny, Annie Tran, Cathy Saiki, Hal Johnson, Kristin Yeoman, Tom Safranek, Kathleen Creppage, Alicia Lepp, Tracy Miller, Nato Tarkhashvili, Kristine E. Lynch, Joanna R. Watson, Danielle Henderson, Megan Christenson, Sarah Dee Geiger

**Affiliations:** 1 Michigan Department of Community Health; 2 New Mexico Department of Health; 3 Division of Population Health, National Center for Chronic Disease Prevention and Health Promotion, CDC; 4 Council of State and Territorial Epidemiologists (CSTE); 5 California Department of Public Health; 6 Florida Department of Children and Families; 7 CDC EIS Officer (Nebraska Department of Health and Human Services); 8 Nebraska Department of Health and Human Services; 9 CDC/CSTE Applied Epidemiology Fellow (North Carolina Division of Public Health); 10 North Dakota Department of Health; 11 CDC Career Epidemiology Field Officer, Office of Public Health Preparedness and Response (South Dakota Department of Health); 12 CDC/CSTE Applied Epidemiology Fellow (Utah Department of Health); 13 CDC EIS Officer (Utah Department of Health); 14 Virginia Department of Health; 15 CDC/CSTE Applied Epidemiology Fellow (Wisconsin Division of Public Health); 16 Wisconsin Division of Public Health

Excessive alcohol consumption, the fourth leading preventable cause of death in the United States (1), resulted in approximately 88,000 deaths and 2.5 million years of potential life lost (YPLL) annually during 2006–2010 and cost an estimated $223.5 billion in 2006 (2). To estimate state-specific average annual rates of alcohol-attributable deaths (AAD) and YPLL caused by excessive alcohol use, 11 states analyzed 2006–2010 data (the most recent data available) using the CDC Alcohol-Related Disease Impact (ARDI) application. The age-adjusted median AAD rate was 28.5 per 100,000 population (range = 50.9 per 100,000 in New Mexico to 22.4 per 100,000 in Utah). The median YPLL rate was 823 per 100,000 (range = 1,534 YPLL per 100,000 for New Mexico to 634 per 100,000 in Utah). The majority of AAD (median = 70%) and YPLL (median = 82%) were among working-age (20–64 years) adults. Routine monitoring of alcohol-attributable health outcomes, including deaths and YPLL, in states could support the planning and implementation of evidence-based prevention strategies recommended by the Community Preventive Services Task Force to reduce excessive drinking and related harms. Such strategies include increasing the price of alcohol, limiting alcohol outlet density, and holding alcohol retailers liable for harms related to the sale of alcoholic beverages to minors and intoxicated patrons (dram shop liability) (3).

The ARDI Custom Data module* was used for this analysis by 11 states (California, Florida, Michigan, Nebraska, New Mexico, North Carolina, North Dakota, South Dakota, Utah, Virginia, and Wisconsin) participating in the Council of State and Territorial Epidemiologists’ Alcohol Subcommittee. ARDI estimates AAD and YPLL resulting from excessive alcohol use by using multiple data sources and methods (4).† ARDI estimates AAD by multiplying the number of age- and sex-specific deaths from 54 alcohol-related conditions by the alcohol-attributable fractions (AAF) for that condition. AAF are used to express the extent to which alcohol consumption contributes to a health outcome. AAF estimate the proportion of deaths from various causes that are directly or indirectly attributable to alcohol consumption. The AAF range from 1.0 for 15 conditions (e.g., alcoholic liver disease and alcoholic polyneuropathy) to as low as 0.01 (e.g., hypertension and hemorrhagic stroke in females). The AAF used in ARDI and for this analysis are provided in the application. YPLL by age, sex, and race/ethnicity were calculated by multiplying age- and sex-specific AAD estimates for each cause by the corresponding life expectancy estimate at the time of death.§ For chronic causes of death (e.g., liver disease), AAD and YPLL were estimated for decedents aged ≥20 years; for acute causes, they were estimated for decedents aged ≥15 years. AAD and YPLL also were estimated for persons aged <15 years who died from motor-vehicle crashes, child maltreatment, or low birth weight. State death certificate data from 2006–2010, the most recent available for participating states, were used to determine the average annual number of alcohol-related deaths for the 54 alcohol-related conditions assessed by the ARDI application and to obtain decedent demographic information. Death records missing data on decedent age, sex, or race/ethnicity were excluded. Prevalence data on alcohol use for 2006–2010 were obtained from state Behavioral Risk Factor Surveillance Systems and used to calculate AAF for most chronic conditions profiled in ARDI. Average annual state rates for AAD and YPLL per 100,000 population for 2006–2010 were calculated by dividing the average annual AAD and YPLL estimates for 2006–2010 by the average annual bridged-race population estimates from the U.S. Census for 2006–2010, and then multiplying by 100,000. The rates were then age-adjusted to the 2000 U.S. population.

During 2006–2010, the median age-adjusted AAD rate was 28.5 per 100,000 (state median AAD = 1,647; rate range = 50.9 deaths per 100,000 in New Mexico to 22.4 per 100,000 in Utah) (Table 1). The median AAD rates increased with age, and the majority of AAD (median 70%) involved working-age (20–64 years) adults. The median AAD rate was highest (60.3 per 100,000) for persons aged ≥65 years and lowest (4.1 per 100,000) for persons aged 0–19 years. The median age-adjusted AAD rate for men (42.4 per 100,000) was more than twice the median age-adjusted AAD rate for women (15.8 per 100,000). AAD rates varied substantially by race and ethnicity; some states (e.g., North Dakota and South Dakota) had very high rates of AAD among American Indians/Alaska Natives (AI/AN), whereas rates in other states (California, Michigan, and Virginia) were highest among blacks (Table 1).

During 2006–2010, the median age-adjusted YPLL rate was 823 per 100,000 population (state median YPLL = 42,756; rate range = 1,534 YPLL per 100,000 in New Mexico to 634 YPLL per 100,000 in Utah) (Table 2). The median YPLL rates were highest among persons aged 35–49 years (state median YPLL = 12,486; median state rate = 1,183 per 100,000) and lowest among persons aged 0–19 years (state median YPLL = 3,285; median state rate = 256 per 100,000). A median of 82% of all alcohol-attributable YPLL involved working-age adults (range = 85% in New Mexico to 78% in Nebraska). The median YPLL rate for men (1,215 per 100,000) was more than twice the median rate for women (456 per 100,000). YPLL rates were highest for AI/AN, ranging from 4,195 YPLL (South Dakota) to 200 YPLL per 100,000 (Virginia) (Table 2).

## Editorial Note

During 2006–2010, excessive alcohol use resulted in a median annual age-adjusted AAD rate of 28.5 per 100,000 population and a median YPLL rate of 823 per 100,000 in the 11 states studied. Approximately two out of three deaths and four out of five YPLL were among working-aged adults, and more than two thirds of AAD and YPLL involved males. Although the majority of AAD involved non-Hispanic whites, the median AAD rate for AI/AN (60.6 per 100,000) was twice as high as the AAD rate for any other racial or ethnic group. These findings are consistent with other published estimates on the distribution of AAD and YPLL by sex (4), disparities by race/ethnicity within states (5), and differences in AI/AN rates among states (6).

What is already known on this topic?

The health consequences of excessive alcohol use vary across geographically diverse states and include substantial disparities in alcohol-related outcomes by sex and race/ethnicity.

What is added by this report?

Adjusted to the 2000 U.S. standard population, in a convenience sample of 11 states, the median alcohol-attributable death (AAD) rate was 28.5 per 100,000, and the median years of potential life lost (YPLL) was 823 per 100,000 during 2006–2010. The majority of AAD (median 70%) and YPLL (median = 82%) were among working-age adults (aged 20–64 years).

What are the implications for public health practice?

Routine monitoring of alcohol-attributable health outcomes, including deaths and YPLL, in states could support the planning and implementation of evidence-based prevention strategies recommended by the Community Preventive Services Task Force to reduce excessive drinking and related harms. Such strategies include increasing the price of alcohol, limiting alcohol outlet density, and holding alcohol retailers liable for harms related to the sale of alcoholic beverages to minors and intoxicated patrons (dram shop liability).

The findings in this report highlight the ongoing public health impact of excessive drinking in the United States, as well as the geographic and demographic disparities in AAD and YPLL. Differences in age-adjusted rates of AAD and YPLL among states probably reflect differences in the prevalence of excessive drinking (7), which is affected by various factors, including state and local laws governing the price, availability, and marketing of alcoholic beverages (8). These death rates also might reflect the influence of other factors (e.g., rurality and access to trauma care) that could affect the risk for death from alcohol-attributable conditions (9). The high rates of AAD and YPLL among working-age adults further highlight the impact of excessive alcohol use throughout a person’s lifespan, and were a major contributor to alcohol-attributable productivity losses from premature mortality that, together with lost wages, were responsible for 72% of the estimated $223.5 billion in economic costs in 2006 (2). The AAD and YPLL rates were lower among the 0–19 years age group because this age group had fewer AAD compared with other age groups.

The findings in this report are subject to at least seven limitations. First, ARDI exclusively uses the underlying cause of death and does not consider contributing causes that might be alcohol-related. Second, ARDI does not include AAD estimates for several causes (e.g., tuberculosis) for which excessive alcohol use is believed to be an important risk factor. Third, the alcohol data used to calculate AAF estimates were based on self-reports and might underestimate the actual prevalence of excessive alcohol use (10). Fourth, state estimates calculated in this study might be different than those available in the ARDI application. Fifth, national AAF data were used, even though studies suggest that there are important state differences in AAF for some causes of alcohol-attributable deaths. Sixth, AAD and YPLL rates could not be calculated for some age and race/ethnicity categories because of the small number of AAD in some of these groups. Finally, some AI/AN might have been misclassified by race on death certificates, which could have resulted in an underestimate of the number of AI/AN deaths and YPLL in states (6).

The Community Preventive Services Task Force has recommended several population-level, evidence-based strategies to reduce excessive drinking and related harms, including increasing the price of alcohol, limiting alcohol outlet density, and holding alcohol retailers liable for harms related to the sale of alcoholic beverages to minors and intoxicated patrons (dram shop liability) (3). Routine monitoring of alcohol-attributable health outcomes, including deaths and YPLL, in states could support the planning and implementation of evidence-based prevention strategies to reduce excessive drinking and related harms.

## Figures and Tables

**TABLE 1:** Average annual alcohol-attributable deaths (AAD)* and rates, by selected characteristics — 11 U.S. states, 2006–2010

	California		Florida		Michigan		Nebraska		New Mexico		North Carolina		North Dakota		South Dakota		Utah		Virginia		Wisconsin	
											
Characteristic	AAD	Rate	AAD	Rate	AAD	Rate	AAD	Rate	AAD	Rate	AAD	Rate	AAD	Rate	AAD	Rate	AAD	Rate	AAD	Rate	AAD	Rate
**Age group (yrs)** †																						
0–19	390	3.7	185	4.1	121	4.4	21	4.2	34	6.0	106	4.2	—**	—**	12	5.4	23	2.5	73	3.5	54	3.6
20–34	1,583	20.1	1,014	29.3	430	23.1	66	17.9	166	41.6	502	27.0	27	18.9	40	25.0	103	15.7	320	19.7	214	19.6
35–49	2,546	31.8	1,451	37.3	709	33.4	95	26.5	289	72.2	669	33.1	45	36.2	60	39.1	124	26.3	448	25.6	352	29.2
50–64	3,398	56.3	1,879	53.6	916	47.4	113	34.5	299	78.0	753	44.0	42	33.4	66	44.2	146	39.6	512	34.8	451	41.9
≥65	2,578	64.8	1,718	54.8	926	70.2	141	58.6	245	94.5	676	57.8	58	60.3	81	70.8	117	49.7	480	51.6	577	76.4
**Sex** §																						
Male	7,589	43.9	4,460	46.3	2,095	42.4	295	33.4	723	73.4	1,930	42.7	123	36.6	175	43.9	354	31.0	1,297	33.7	1,092	38.5
Female	2,906	15.8	1,788	16.6	1,006	18.1	140	14.6	310	29.4	777	15.4	56	15.8	83	19.4	158	13.9	535	12.7	555	17.7
**Race/Ethnicity** § ¶																						
AI/AN	129	25.4	17	20.2	29	40.7	12	65.4	182	99.2	47	35.2	36	122.8	74	133.2	19	60.6	—**	—**	32	61.4
A/NH/PI	589	11.9	40	8.3	21	11.0	—**	—**	—**	—**	15	8.8	—**	—**	—**	—**	—**	—**	32	8.6	14	15.1
Black	913	36.6	725	25.4	594	42.9	24	29.7	16	31.8	578	29.3	—**	—**	—**	—**	—**	—**	388	25.4	121	39.0
White, Hispanic	3,013	33.4	792	22.0	44	16.3	20	19.9	409	53.3	109	20.5	—**	—**	—**	—**	50	25.4	68	16.4	46	26.4
White, non-Hispanic	5,775	31.2	4,613	35.2	2,342	27.4	372	22.7	411	40.2	1,953	28.6	139	21.4	178	23.4	430	21.9	1,338	23.5	1,433	27.0
**Total** §	**10,495**	**29.4**	**6,248**	**31.0**	**3,102**	**29.9**	**436**	**23.7**	**1,033**	**50.9**	**2,707**	**28.5**	**179**	**26.2**	**259**	**31.5**	**513**	**22.4**	**1,832**	**22.8**	**1,647**	**27.9**

**Abbreviations:** AAD = alcohol-attributable deaths; AI/AN = American Indian/Alaska Native; A/NH/PI = Asian, Native Hawaiian, or Pacific Islander.

* The CDC Alcohol-Related Disease Impact application estimates AAD resulting from excessive alcohol use by using multiple data sources and methods. Additional information on the methods is available at http://apps.nccd.cdc.gov/dach_ardi/info/methods.aspx.

† Rates are age-specific per 100,000 population.

§ Rates are per 100,000 population, age-adjusted to the U.S. 2000 standard population.

¶ Non-white Hispanics are included in the other racial groups.

** Race/ethnicity estimates <10 are suppressed.

**TABLE 2:** Average annual alcohol-attributable years of potential life lost (YPLL)* and rates, by selected characteristics — 11 U.S. states, 2006–2010

	California		Florida		Michigan		Nebraska		New Mexico		North Carolina		North Dakota		South Dakota		Utah		Virginia		Wisconsin	
											
Characteristic	YPLL	Rate	YPLL	Rate	YPLL	Rate	YPLL	Rate	YPLL	Rate	YPLL	Rate	YPLL	Rate	YPLL	Rate	YPLL	Rate	YPLL	Rate	YPLL	Rate
**Age group (yrs)** †																						
0–19	23,736	227	11,124	247	7,565	278	1,300	256	2,106	368	6,520	260	436	256	747	333	1,427	154	4,479	217	3,285	218
20–34	79,511	1,009	51,066	1,475	21,537	1,159	3,316	905	8,281	2,073	25,271	1,357	1,365	950	1,990	1,258	5,149	784	16,199	999	10,782	986
35–49	89,917	1,123	51,528	1,324	25,161	1,185	3,399	949	10,285	2,573	23,903	1,183	1,627	1,298	2,139	1,383	4,468	944	15,945	911	12,486	1,035
50–64	80,709	1,338	44,611	1,271	21,874	1,132	2,665	817	7,148	1,867	17,872	1,044	984	790	1,579	1,061	3,497	951	12,137	824	10,732	999
≥65	27,187	684	17,495	558	9,250	702	1,368	568	2,538	981	7,143	611	570	595	790	695	1,220	518	4,943	531	5,470	724
**Sex** §																						
Male	221,055	1,215	126,524	1,388	59,769	1,220	8,373	940	21,508	2,201	58,658	1,285	3,520	1,057	5,038	1,277	11,027	875	38,794	986	29,662	1,048
Female	80,005	434	49,299	510	25,618	493	3,676	410	8,851	878	22,050	457	1,462	456	2,207	561	4,733	392	14,908	363	13,094	447
**Race/Ethnicity** § ¶																						
AI/AN	4,013	691	569	599	905	1,159	428	2,060	6,350	3,194	1,722	1,170	1,288	3,893	2,637	4,195	673	1,794	85	200	1,069	1,819
A/NH/PI	16,312	309	1,254	237	658	271	97	267	160	438	545	251	—**	—**	28	320	225	269	935	211	473	398
Black	31,451	1,187	26,269	849	20,566	1,411	973	1,062	548	1,037	19,370	939	56	940	80	700	188	694	13,041	809	4,385	1,227
White, Hispanic	99,827	915	25,407	668	1,562	475	802	625	12,714	1,564	4,779	705	35	463	127	858	1,894	728	2,706	516	1,698	713
White, non-Hispanic	146,958	858	120,193	1,072	59,380	742	9,561	627	10,299	1,157	54,074	850	3,543	586	4,354	622	12,752	617	36,786	680	35,097	708
**Total** §	**301,060**	**823**	**175,824**	**944**	**85,387**	**853**	**12,049**	**675**	**30,358**	**1,534**	**80,708**	**863**	**4,982**	**763**	**7,245**	**923**	**15,760**	**634**	**53,703**	**670**	**42,756**	**748**

**Abbreviations:** YPLL = years of potential life lost; AI/AN = American Indian/Alaska Native; A/NH/PI = Asian, Native Hawaiian, or Pacific Islander.

* The CDC Alcohol-Related Disease Impact application estimates YPLL resulting from excessive alcohol use by using multiple data sources and methods. Additional information on the methods is available at http://apps.nccd.cdc.gov/dach_ardi/info/methods.aspx.

† Rates are age-specific per 100,000 population.

§ Rates are per 100,000 population, age-adjusted to the U.S. 2000 standard population.

¶ Non-white Hispanics are included in the other racial groups.

** Race/ethnicity estimates <10 are suppressed.

## Footnotes

* Available at http://apps.nccd.cdc.gov/ardi.

† Additional information available at http://apps.nccd.cdc.gov/dach_ardi/info/methods.aspx.

§ Available at http://www.cdc.gov/nchs/products/life_tables.htm#life for 2006–2009, and at http://www.cdc.gov/nchs/data/nvsr/nvsr61/nvsr61_04.pdf for 2010.
